# Experimental comparison of X-ray ptychographic and holographic nanotomography of metal-stained neuronal tissue

**DOI:** 10.1364/OE.563186

**Published:** 2025-06-30

**Authors:** Alexandra Pacureanu, Thomas Thies, Carles Bosch, Mirko Holler, Manuel Guizar-Sicairos, Elisabeth MÜLler, Joakim Reuteler, Dmitry Karpov, Andreas T. Schaefer, Peter Cloetens, Andreas Menzel, Ana Diaz

**Affiliations:** 1 ESRF, the European Synchrotron, 71 Av. des Martyrs, 38000 Grenoble, France; 2 Paul Scherrer Institute, Forschungsstrasse 111, 5232 Villigen-PSI, Switzerland; 3 Francis Crick Institute, 1 Midland Rd, London NW1 1AT, United Kingdom; 4 École Polytechnique Fédérale de Lausanne (EPFL), 1015 Lausanne, Switzerland; 5 ScopeM, ETH Zurich, Switzerland

## Abstract

Hard X-ray nanotomography is a promising technology for nondestructive imaging of biological tissues with three-dimensional isotropic resolution. The implementation of fourth-generation synchrotron sources brings coherent X-ray microscopy to the central stage and fosters further development of this class of techniques. Here, we present an experimental comparison of X-ray near-field ptychography and X-ray holography, two high-resolution X-ray microscopy techniques applied under cryogenic conditions to the exact same sample at two different synchrotron sources. Using a heavy-metal-stained, resin-embedded brain tissue sample, we obtain similar contrast and spatial resolutions at equivalent radiation doses with these two approaches. We discuss the current benefits and limitations of the two methods. These results provide a basis for developments in X-ray microscopy of biological samples at present and future beamlines of fourth-generation synchrotron sources.

## 1 Introduction

Hard X-ray microscopy (HXM) offers a non-destructive approach to imaging biological tissues with thicknesses ranging from micrometers to millimeters with X-ray photon energies typically ranging from 5 keV to beyond 100 keV. Challenges in the implementation of such technologies include the design and fabrication of high-precision X-ray optics and actuators, high-sensitivity and high-resolution detectors, and, specific to soft biological tissues, mitigation of the low-absorption contrast and sensitivity to radiation dose. For the latter, imaging under cryogenic conditions may delay the radiation damage both in biological [1,2] and polymer samples [3]. In order to overcome these challenges, several experimental approaches have been proposed. Zernike phase contrast X-ray microscopes have been used both at room temperature to image biological tissues [4], and in combination with a cryo-jet to image frozen hydrated yeast cells [5]. However, due to the limited efficiency of the magnifying lens, which is placed downstream of the sample, this approach requires higher radiation doses than other techniques, as only about 10% of the X-rays passing through the lens actually contribute to image formation. X-ray holography served for imaging *Deinococcus radiodurans*, a particularly radiation-resistant bacterium, in 2D at room temperature [6]. On the other hand, ptychographic X-ray computed tomography (PXCT) has been used to image frozen hydrated *Chlamydomonas* unicellular algae, either using a cryo-jet [7] or an in-vacuum chamber with a cryogenic sample stage [8]. Published calculations show that, in principle, X-ray holography and X-ray ptychography should be equally dose efficient in such a way that in all cases one should be able to achieve comparable resolution for the same X-ray dose [9].

There are several differences between X-ray holography and X-ray ptychography, or between their corresponding extensions to 3D imaging, called X-ray holographic nanotomography (XNH) and ptychographic X-ray computed tomography (PXCT). X-ray holography operates in full-field mode and uses longitudinal diversity by acquiring data at several effective propagation distances, typically four, in order to reconstruct phase images [10,11]. On the other hand, in X-ray ptychography the illumination is typically smaller than the reconstructed field of view, the sample position along the beam direction does not change, and the sample is scanned in the plane perpendicular to the beam direction at many different positions, effectively using transverse diversity [12]. Ptychographic phase-retrieval algorithms are then used to reconstruct the complex-valued transmission function of the sample [13]. The instruments designed and optimized for either technique can be substantially different. In PXCT, achieving high resolution crucially depends on precisely positioning the sample throughout the entire field of view, which can be many tens of microns, with step sizes of a few microns and an accuracy better than the target resolution, ideally a few tens of nanometers. The OMNY instrument [14] operating at the cSAXS beamline of the Swiss Light Source in Villigen, Switzerland, has been optimized for such measurements under cryogenic conditions [15–17]. X-ray ptychography is often used in far-field geometry, where coherent diffraction patterns are recorded several meters downstream of the sample using detectors optimized for X-ray scattering, featuring single photon counting capability to detect the weak scattering at large angles and a physical pixel size typically between 50 and 200 *µ*m [18–20]. In the case of XNH, typically sCMOS or CCD sensors with pixel sizes in the range of ten to twenty microns are coupled to scintillators for indirect detection, as in this case the pixel size can be a limiting factor for resolution. The endstation at the ID16A nano-imaging beamline of the European Synchrotron in Grenoble, France [21,22], is a state-of-the-art instrument optimized for XNH measurements [23–25]. The different experimental implementations of PXCT and XNH raise the question of whether one of them is more dose efficient than the other, or how they compare in terms of resolution, data quality and measurement time. This is relevant for making informed decisions regarding the construction of future instruments for high-resolution X-ray microscopy on biological tissues in fourth-generation synchrotron storage rings [26–28].

Here we present a comparison between PXCT and XNH as two approaches to HXM for imaging metal-stained brain tissue in 3D. We conducted measurements on the exact same specimen to ensure identical density contrast. We employed instruments optimized for each technique by measuring the sample at two different synchrotron facilities. In earlier work, metal stained, resin-embedded biological tissues have been measured at cryogenic temperature with hard X-ray microscopy after showing severe shrinkage and deformation when imaged at room temperature [29]. Moreover, this type of samples are expected to exhibit structural changes when warmed up to room temperature after being exposed to high radiation dose under cryogenic temperatures. Therefore, in our study we acquired first near-field PXCT data at the OMNY instrument at the cSAXS beamline in Switzerland, and later collected XNH data at the ID16A beamline in France on the same metal-stained, resin-embedded mouse brain sample, while keeping the specimen at cryogenic temperature during the whole process. We explain the sample choice and describe its preparation in Section 2. Experimental details for the cSAXS and ID16A measurements are given in Sections 3 and 4, respectively. Section 5 then compares the two measurements, followed by discussion (Section 6) and conclusions (in Section 7).

## 2 Sample description

The selected sample is heavy-metal-stained mouse brain tissue cut into a cylindrical shape. Brain tissue features a dense hierarchical structure spanning different length scales, from several micron-sized neuronal cell bodies and dendrites to subcellular structures measuring hundreds or tens of nanometers. These features have been well characterized by volume electron microscopy (EM) [30], which serves as ground truth for evaluating X-ray nanotomography. We used sample preparation protocols that have been developed for EM, including tissue fixation, heavy-metal staining, and resin embedding, which preserve the ultrastructure of the tissue over large volumes, e.g. for connectomics [31].

Mouse brain tissue of an adult (13.2 week-old) male mouse of C57/Bl background (sample C319) was fixed, stained, and embedded as previously described [32]. Briefly, after cervical dislocation, the brain was extracted and manipulated in cold dissection buffer (4.6% sucrose, 0.563 mM CaCl_2_, 65.2 mM NaH_2_PO_4_·H_2_O with 0.02% sodium azide, bubbled with 95% O_2_/5% CO_2_). Shortly after (*<*10 min), a 600 *µ*m-thick dorsal slice (~ 3 × 3 mm long and wide) of the olfactory bulb was cut out with a vibratome (Leica VT1200) and transferred to cold fixative (1% glutaraldehyde in 0.15 M sodium cacodylate buffer, pH 7.4, with 2 mM CaCl_2_ and 0.02% sodium azide) and fixed overnight at 4 °C. After washing out the fixative with wash solution (0.15 M sodium cacodylate pH 7.4 with 2 mM CaCl_2_ and 0.02% sodium azide), the tissue was sequentially stained with heavy metals and washed with distilled water: reduced osmium tetroxide (2% OsO_4_, 3% potassium ferrocyanide, 2 mM CaCl_2_ in 0.15 M sodium cacodylate buffer pH 7.4, 1 h at 4 °C), wash 5 × 10 min at 20 °C and 1 × 10 min at 50 °C, thiocarbohydrazide (1% aq, 1 h at 50 °C), wash 2 × 10 min at 50 °C and 4 × 10 min at 20 °C, osmium tetroxide (2% OsO_4_ aq, 1 h at 20 °C), wash 6 × 10 min at 20 °C, uranyl acetate (2% aq, overnight at 4 °C), wash 4 × 10 min at 20 °C, lead aspartate (prepared as in Ref. [33], pH 5.5, 1 h at 60 °C), and washed 5 × 10 min at 20 °C. The sample was then dehydrated following a graded series of ethanol incubations: ethanol 70% (1 × 10 min, 20 °C), 90% (2 × 20 min, 20 °C), 100% (2 × 10 min and 1 × 1 h, 4 °C) followed by propylene oxide (2 × 10 min, 20 °C). At this point, the sample was infiltrated with hard epon resin [34] through sequential incubations with resin dissolved in propylene oxide: 25% resin (2 h, 20 °C), 50% resin (2 h, 20 °C), 75% resin (2 h, 20 °C), 100% resin (1 × 2 h, 20 °C and overnight, 20 °C) after which point the sample was transferred to individual silicon moulds and cured (72 h, 60 °C). The sample was examined for staining quality with a laboratory-based micro-CT (Zeiss Versa 510). A ~ 2 × 2 × 0.6 mm^3^ region was trimmed and glued onto an aluminium pin ensuring that the vibratome-cut ventral surface was flush against the pin surface. This left the dorsal plane exposed at the top. The pin was further trimmed to ~ 1 × 1 × 0.6 mm^3^ with conventional ultramicrotomy tools and the blockface was milled with glass and diamond knives (Trim90, Diatome). A graded milling of the sample sides allowed the creation of an open edge, exposing neuropil of the external plexiform layer—a tissue region rich in neurites of multiple sizes and with few cell bodies and, therefore, nuclei.

A specimen from the external plexiform layer (EPL) of approximately 108 µm average diameter and 200 µm height was then milled using a Xe-plasma focused ion beam (FIB) instrument at ScopeM, ETH Zürich, Switzerland. The sample was subsequently transferred to a Ga FIB at the PSI in Villigen, Switzerland, to perform an undercut and a lift-out process for mounting onto an OMNY pin [35].

## 3 Measurement with ptychographic X-ray computed tomography

Ptychographic imaging was performed at the cSAXS beamline of the Swiss Light Source (SLS) at the Paul Scherrer Institute in Villigen, Switzerland, at a photon energy of 6.2 keV. We used the tOMography Nano crYo stage (OMNY), operating in ultra-high vacuum and designed for PXCT measurements of high reliability and high spatial resolution [14]. Although the sample preparation protocol did not include cryogenic fixation, the sample was kept at low temperature during imaging to avoid deformation and resin mass loss induced by X-rays [3,29,36]. Thus, the specimen was inserted into the OMNY vacuum chamber while at room temperature, then gradually cooled down to approximately 110 K, i.e., the sample storage temperature within OMNY. Subsequently, it was mounted onto the cryogenic stage, which was held at a stable temperature of 88 K throughout alignment and data acquisition.

To efficiently achieve our targeted spatial resolution of approximately 100 nm within a reasonable measurement time—given the available coherent flux at the SLS before its upgrade—we opted for a near-field geometry. While we employed the standard experimental setup commonly used for far-field ptychography at cSAXS, the near-field approach [37,38] allowed us to maximize imaging efficiency without additional hardware modifications. This choice is discussed in more detail in Section 6.

**Fig. 1:**
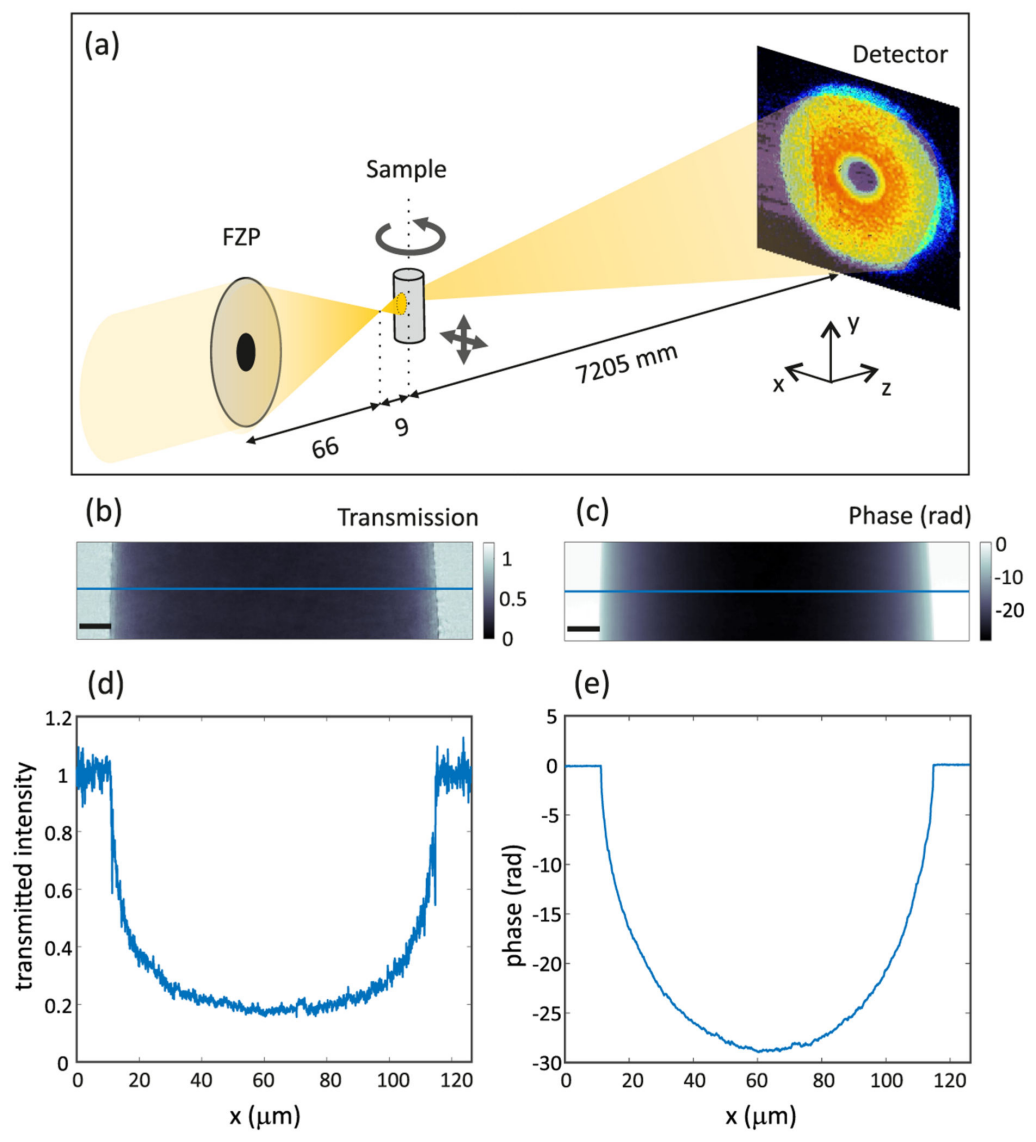
(a) Experimental setup for ptychographic X-ray computed tomography (PXCT) measurements, where a Fresnel zone plate (FZP) is used to define the illumination onto the sample. (b) Transmission and (c) phase image obtained by X-ray ptychography at one projection angle. The scale bar is 10 µm. (d,e) Line cuts shown with a blue line in (b) and (c), respectively.

A coherently illuminated Fresnel zone plate (FZP) of 220 µm diameter and 60 nm outermost zone width defined a coherent illumination on the sample, as shown in Fig. 1(a). With this configuration, the focal length of the FZP was 66 mm and the focused beam had a coherent flux of 7 × 10^8^ photons/s. The flux was measured with the same detector used for data collection, which is described below. The FZP featured locally displaced zones, with a design to provide an optimized illumination for ptychography [39]. We placed the sample 9 mm downstream of the focus, where the illumination was about 30 µm in diameter. For the acquisition of 2D projections, we scanned the sample in steps of about 4 µm following the pattern of a Fermat spiral [40] with a field of view of 125 µm × 30 µm (horizontal × vertical), resulting in about 234 scan positions. At each scan position, we recorded a coherent diffraction pattern with a 0.1 s exposure time, using an in-vacuum Eiger 1.5M detector (75 µm pixel size) [18] placed 7.205 m from the sample. For tomographic acquisitions, we repeated ptychographic acquisitions at 1500 equally spaced rotation angles of the sample around the vertical axis, as shown in Fig. 1(a). The angular step sequence was non-sequential: we first acquired a tomogram with double angular step size, followed by a second tomogram using the same angular step size but with all angles interleaved to achieve uniform angular sampling. Such binary decomposition with 2 sequential sparse subtomograms [41] is useful for assessing any changes in the sample during acquisition, confirming that the sample did not undergo any structural changes or radiation damage due to the X-ray exposure. The total acquisition time was 15 h 45 min, including exposure time and overhead due to the movement of scanning and rotation stages between acquisitions. We estimate that a dose of (3.3 ± 0.5) × 10^7^ Gy was imparted to the sample for the acquisition (see Appendix). In our near-field ptychographic reconstructions, the strong curvature of the incident beam is taken into account by an appropriate near-field propagation of the wavefront from the sample to the detector, which uses two Fourier transforms [37]. Ptychographic reconstructions were performed in real time during the experiment using PtychoShelves [42] with 1500 iterations of the difference map algorithm [43] followed by 300 iterations of a GPU-based maximum likelihood algorithm [44] implemented with compact partitions as described in Ref. [45]. Experimental parameters, namely the distance from the focus to the sample, the distance from the sample to the detector, and the detector pixel size, set the reconstructed image pixel size to 93.8 nm. In Figure S1 of the Supplement 1 we show an example of the illumination which is reconstructed simultaneously with the sample image. Because the horizontal field of view of the ptychographic scans was larger than the sample, we also reconstructed some empty area on both sides of the sample. This allowed us to normalize both the transmission image, computed as the square of the amplitude, and the image of the phase shift of the X-rays as they propagate through the sample, as shown in Figs. 1(b) and 1(c), respectively. The spatial resolution of the 2D phase image was estimated by Fourier ring correlation between two images acquired at the same angle using the 1-bit threshold criterion [46], resulting in ~ 102 nm. Phase images computed at all tomographic angles were further aligned with sub-pixel resolution [29,47] and reconstructed tomographically by filtered back-projection with a ramp filter. This yields the 3D electron density distribution of the specimen with absolute quantitative contrast [48]. The 3D spatial resolution of the dataset was estimated via Fourier shell correlation of the two subtomograms using the 1/2-bit threshold [46], as detailed in Section 5.

After the PXCT measurements, the sample was unloaded from the OMNY instrument under vacuum using a Leica VCT100 load-lock system and immediately immersed in liquid nitrogen. This process ensured that the sample remained below 110 K throughout the transfer. Once in liquid nitrogen, the sample was transferred with tweezers from the OMNY sample transfer block to a custom-made cryo-storage container [35], and then stored in a dry shipper for delivery to ESRF.

## 4 Measurement with X-ray holographic nanotomography

**Fig. 2:**
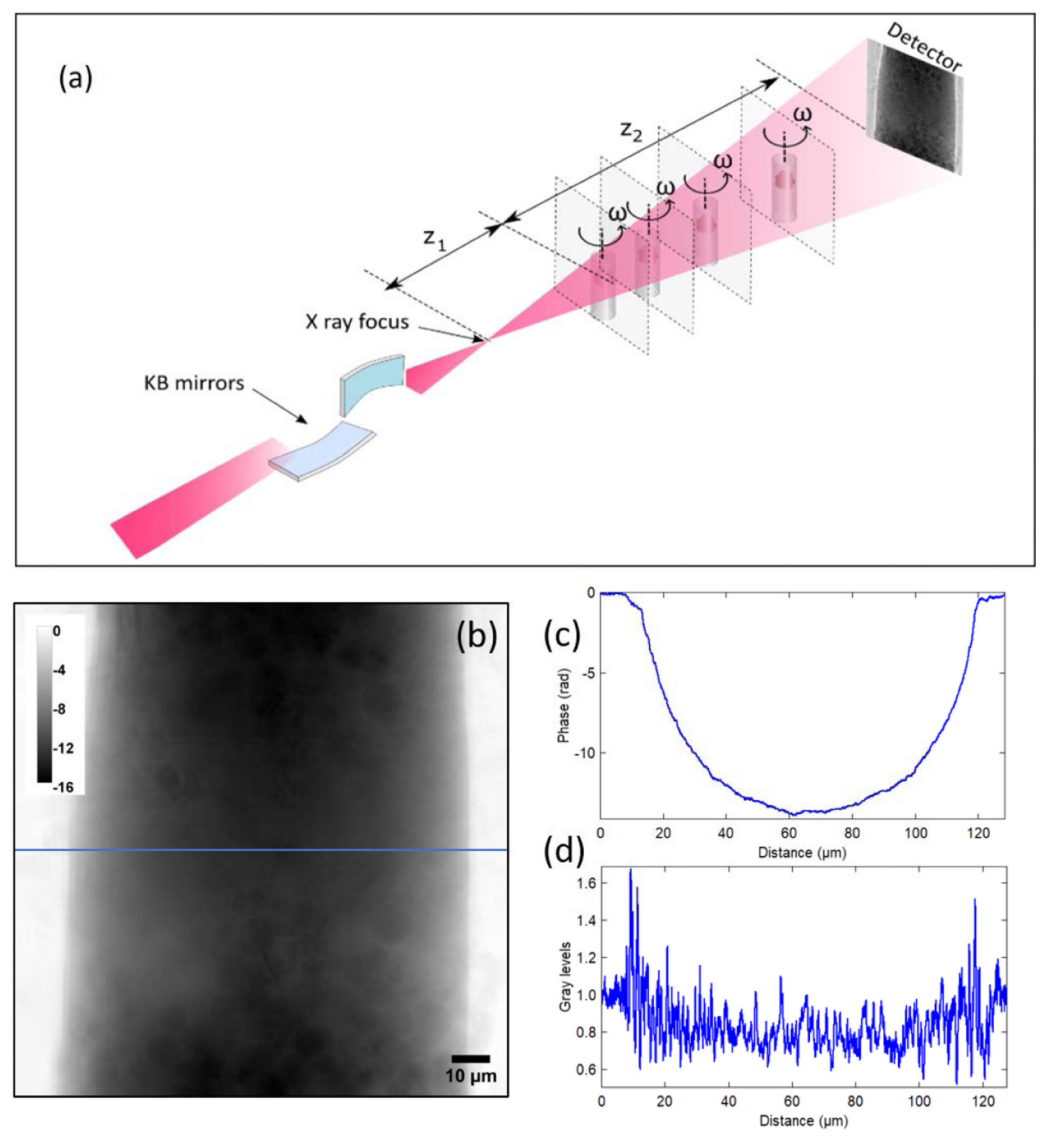
(a) Experimental setup for X-ray holographic nanotomography (XNH) measurement, where Kirkpatrick-Baez (KB) multilayer mirrors are used to define the illumination onto the sample. For each angular position, acquisitions at 4 different distances of the sample from the focus, *z*_1_, and to the detector, *z*_2_, were performed. (b) A reconstructed phase map using the four acquired propagation distances. The color bar represents phase in radians. (c) Plot along the line seen in panel (b). (d) Line plot across a flat field corrected raw projection

XNH measurements took place at the ID16A beamline of the ESRF in Grenoble, France. The sample, maintained at cryogenic temperature after imaging at SLS, was transferred from the dry shipper to the Leica VCM cryo-loading station with minimal exposure to air to reduce ice accumulation on the surface. Nevertheless, the long-term storage in a Dewar caused some ice formation, as seen in Fig. 2(b). The sample was then loaded into the ID16A endstation under cryogenic conditions. The setup used for XNH measurements is described in Refs. [21,22]. A beam focused by Kirkpatrick-Baez (KB) mirrors with a total flux of 1.1 × 10^11^ photons/s was used for illuminating the sample. The ID16A beamline uses a single bounce multilayer monochromator (B4C/[Ru/B4C]42/Cr) and the KBs are also multilayer coated (WB4C), resulting in a bandwidth of 10^−2^. The photon flux was measured with a calibrated diode. A schematic of the setup is shown in Fig. 2(a). The sample was measured at 17 keV and maintained at 110 K. Four tomographic scans with 2000 projections each were recorded at different distances from the focus to the sample, corresponding to *z*_1_ values in Fig. 2(a) of 16.1 mm, 16.8 mm, 19.6 mm, and 25.3 mm. At each angular position, the sample was slightly shifted laterally to correct for wavefront inhomogeneities [49]. The exposure time per projection was 150 ms. Supplement 1 Figure S2 includes the reconstructed amplitude and phase of the probe that was used for the acquisition. The detector used was composed of a Frelon CCD sensor with a physical pixel size of 15 *µ*m coupled with a GGG:Eu 23 *µ*m-thick scintillator, resulting in a pixel size of 3 *µ*m after optical magnification. The optical transfer function of the detector at Nyquist frequency is 0.55. The efficiency of the detector at this energy is about 93%. We estimate that a dose of (8.6 ± 0.5) × 10^7^ Gy was imparted on the specimen during the acquisition (see Appendix).

The holograms recorded at different propagation distances were aligned using cross correlation and combined to generate phase maps through an iterative algorithm starting from a first approximation obtained with a multi-distance Paganin approach [50]. The material-specific regularization parameter was set to 9, which corresponds to the *δ* / *β* ratio of Os at 17 keV. Figure 2(b) illustrates an example of a reconstructed phase projection with the profile across the sample width displayed in Fig. 2(c). The reconstructed pixel size is 40 nm. The angular set of phase images was used for tomographic reconstruction using filtered back-projection, resulting in a cylinder-shaped volume of 128 *µ*m diameter and height.

## 5 Comparison between ptychographic computed tomography and holographic nanotomography

Table 1 compares key experimental parameters for the PXCT and XNH acquisitions, including flux density on the sample, measured volume, voxel size, acquisition time, measurement time, and estimated radiation dose. For the estimation of the flux density, the total fluence, and the dose, the measured flux of the incoming beam was used, as reported in Sections 3 and 4 for the PXCT and the XNH measurements, respectively. The total processing time of the data to obtain the final tomograms is also reported in the table. In the case of the PXCT dataset, the ptychographic reconstructions were processed during the data acquisition. Therefore, the total processing time equals the acquisition time, plus 1 hour to perform the tomogram after the acquisition. We note that the processing time can be accelerated by the use of more GPU’s, which was not necessary in this case, as the processing time was sufficient to keep up with the measurement time. In the case of XNH, the processing was performed after the measurement. The total duration includes the alignment of holograms, phase retrieval and tomographic reconstruction. The typical processing time is around three hours. The phase retrieval is the most time consuming step and it could be accelerated by using more compute nodes.

In the following we present an analysis to compare the two datasets acquired by PXCT and by XNH in terms of data quality and resolution. For this purpose, we cropped both 3D datasets to approximately the same volume of 32 × 32 × 12 *µ*m^3^. The volumes do not match exactly because the datasets differ by several degrees rotation around the vertical axis (*y* axis in Fig. 1), due to slight differences in sample orientation upon mounting. We do not attempt to correct this rotation computationally in order to avoid artifacts in the data that could be introduced by interpolation and that may influence their comparison.

**Fig. 3:**
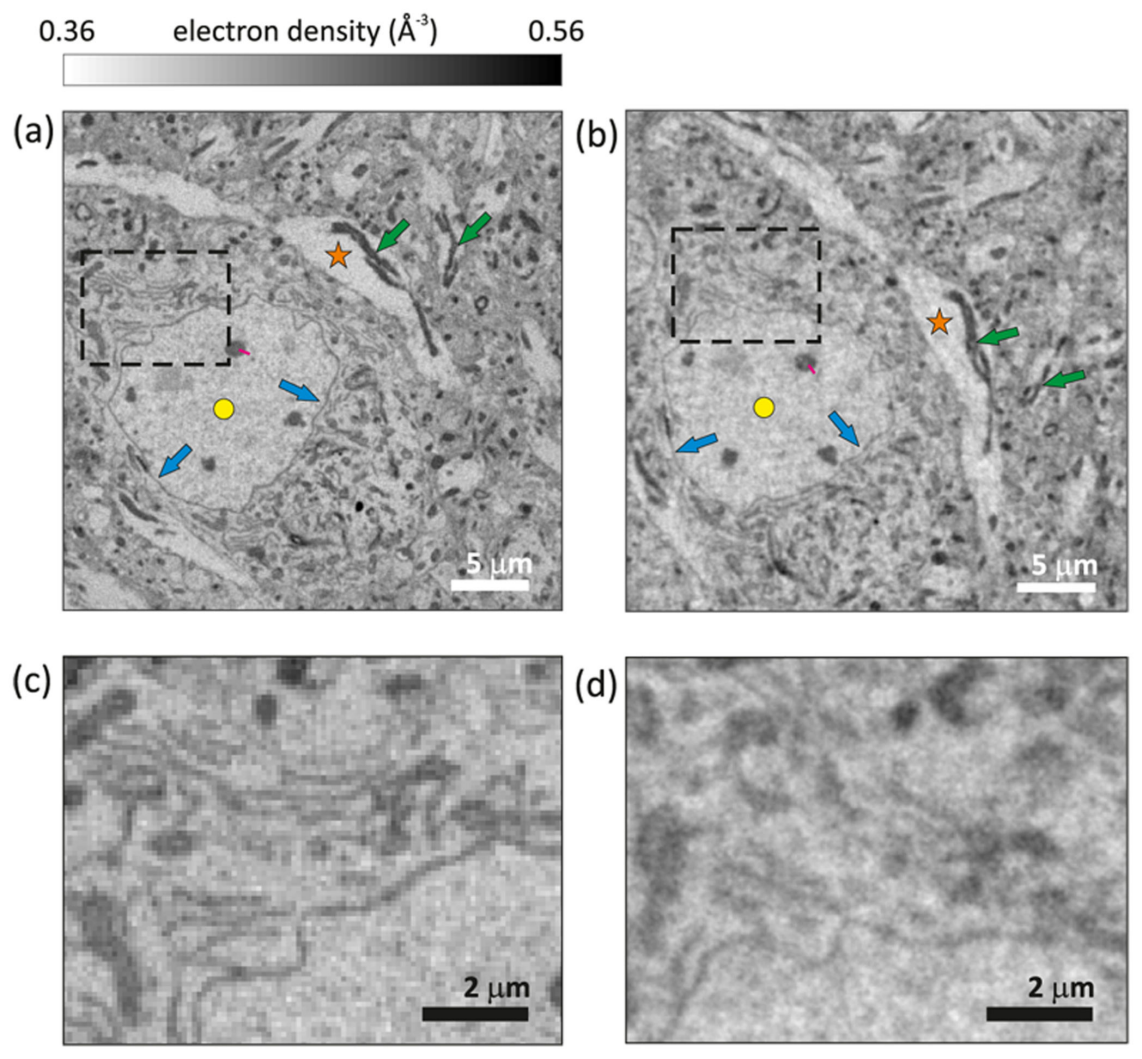
Detail of a slice through the 3D data obtained (a) by PXCT with quantitative electron density contrast indicated by the gray bar and (b) by XNH, showing approximately the same part of the sample. The orange star, yellow dot, blue arrows and green arrows indicate a dendrite, a cell nucleus, a nuclear membrane, and mitochondria, respectively. The black dashed rectangles show areas containing endoplasmatic reticulum structures. (c) and (d) display the enlarged areas within the black dashed rectangles in (a) and (b), respectively.

Figures 3(a) and 3(b) show 2D sections through the PXCT and XNH 3D datasets, respectively, extracted from approximately the same region of the sample. Both slices are perpendicular to their respective rotation axes, which we assume coincide closely, though a slight difference of ≲ 0.1° from different sample mounting cannot be excluded. In both images we observe brain tissue structures, including large features such as dendrites (orange star), a cellular nucleus (yellow dot), and mitochondria (green arrows), but also fine structures such as the nuclear membrane (blue arrows) and the endoplasmic reticulum (ER, dashed rectangle). These features can be identified in both datasets, validating both methods to be suitable for the visualization of subcellular structures in tissues. Some features are remarkably well reproduced with both techniques, like the nuclear membrane indicated by the blue arrows. However, other features are better resolved by PXCT, such as the membrane structures inside the dashed black rectangles, corresponding to the ER. These areas are magnified for clarity in Figs. 3(c) and (d).

**Fig. 4:**
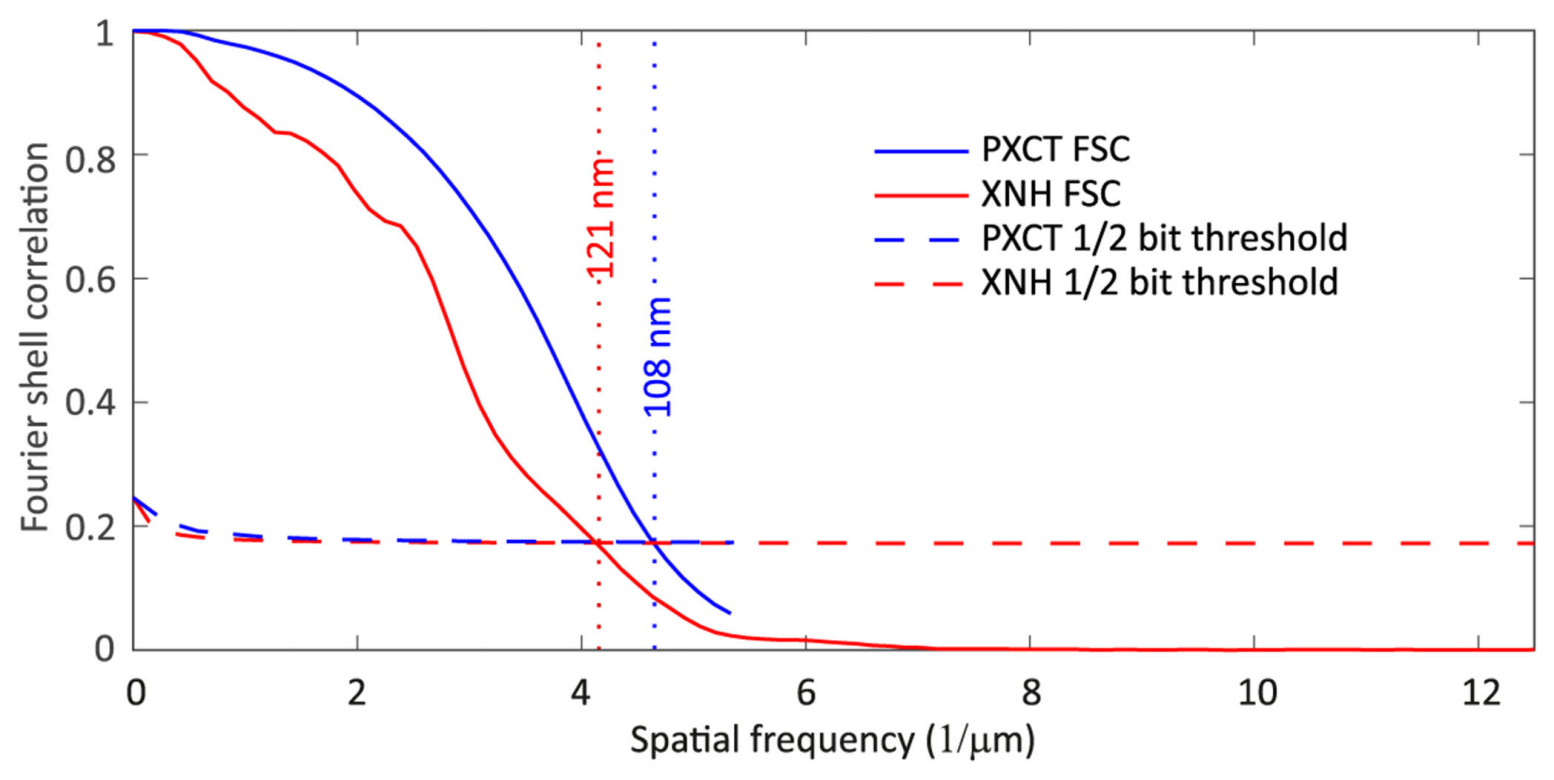
Fourier shell correlation (FSC) analysis to estimate spatial resolution.

To estimate the resolution of both datasets, we used Fourier shell correlation (FSC) [46] computed based on two interleaved subsets of angular projections. In the case of the PXCT dataset, the two subtomograms are sequentially acquired in time, as explained in section 3, whereas in XNH all angular projections are acquired during the same rotation. We then compared the FSC curves to the 1/2-bit threshold criterion [46], corresponding to the half-pitch resolution at a signal-to-noise ratio (SNR) of 0.5 for the full tomographic dataset. Figure 4 shows the resulting FSC curves for both the PXCT and the XNH datasets as a function of spatial frequency. This results in resolution estimates of approximately 108 nm for the PXCT dataset and 121 nm for the XNH dataset, consistent with the visual comparison of slices through the 3D datasets shown in Fig. 3.

## 6 Discussion

In the following we discuss the results obtained for this case study of a single sample. The reconstructed PXCT slices resolve fine structural features, such as membranes, more clearly than the XNH slices, as shown in Fig. 3(c) and (d). Before the XNH measurement, the sample had already been exposed to an estimated dose of 6.6 × 10^7^ Gy, corresponding to two PXCT measurements as described in section 3. Based on previous acquisitions with both techniques [23,36], we expect that the ultrastructure remains unaltered after applying this level of radiation dose. This remains valid as well for the total accumulated dose after the XNH measurement, which was on the order of 1.5 × 10^8^ Gy, a level below the threshold of 6.8 × 10^8^ Gy reported to cause mass loss in similarly prepared samples [36]. However, in the case of XNH, the sample deformed during imaging and there were ice crystals accumulated on the surface. A residual deformation of around 120 nm can be observed when analyzing the aligned projections recorded at zero degrees, before and after the first tomographic scan. This uncorrected deformation causes blurring in the final XNH reconstructed data. In contrast, no deformation was observed during the PXCT measurement.

The results show that the PXCT dataset has slightly better spatial resolution and was acquired with a lower dose compared to XNH. This dose-efficient PXCT measurement is a consequence of a careful choice of experimental parameters based on the 2D resolution achieved in each projection while being aware of the resolution limits imposed by the experimental implementation. In the case of XNH, the main limiting factor for spatial resolution for this measurement is the sample deformation that is not corrected for during the image reconstruction process. More generally, for XNH the spatial resolution is limited by the incoherent contributions of the beam focus. The focus size for this experiment was about 30 nm [21], thus not a limiting factor. The ice particles that appear in the reconstructed phase projection for XNH (Fig. 2(b)), were not there in the phase projection reconstructed by near-field ptychography (Fig. 1(c)). Their presence may induce artifacts in the reconstructed images. For PXCT, the separated reconstruction of the phase and the amplitude of both the probe and the object results in more precise phase retrieval. Nevertheless, the similarity between the two data sets in terms of image quality, resolution, and dose is remarkable considering the different experimental approaches.

Compared to PXCT, measurements with XNH are around 300 times faster in terms of voxels per second and about 16 times faster in terms of resolution elements (resels) per second, as measured by Fourier shell correlation. In theory, when the X-ray attenuation length of the material, *µ*^−1^, matches the sample thickness, fewer photons are needed to achieve a certain image resolution [51], allowing in principle a faster measurement. In this case, neither measurement was performed under optimal conditions. If at all, the PXCT measurement had a closer match of *µ*^−1^ to the average sample diameter of 108 *µ*m compared to the XNH measurement, as shown in Table 1. The main elements that drive differences in acquisition speed here are movements of actuators and photon flux. In the PXCT measurements, the photon flux was lower because of the cSAXS beamline’s optical configuration with a double-crystal monochromator, resulting in a bandwidth of ~ 10^−4^. Moreover, the FZP used to define the illumination on the sample had an efficiency of only about 10%. Independent of the photon flux, the measurement time is limited by overhead associated with actuator movement between scan points of each 2D projection. For this reason, full-field techniques, such as XNH, require fewer movements of the sample and will remain faster. For far-field X-ray ptychography, on-the-fly scan approaches can be used to reduce the overhead time at the expense of very high data rates and computational load [52–54]. In the case of XNH, the main source of overhead time comes from moving the piezo-actuators laterally for each rotation angle [49].

The choice to measure PXCT in near-field geometry was motivated by practical considerations. Aiming for a certain resolution determines the fluence, i.e., the number of photons per unit area, required to achieve that resolution. One way to adjust the fluence would be to reduce the acquisition time at each scan position. However, due to limitations in the scanning frequency, adjusting the average step size is a more convenient way to set the fluence, as lowering the acquisition time would result in a larger total overhead. The average step size determines the total number of scan points and thus the fluence on the sample. In our case, the most efficient scanning parameters to obtain 100 nm resolution in our sample were a step size of 4 *µ*m and an acquisition time of 0.1 s. In our experience, the step size in a ptychographic scan should not exceed a third of the illumination diameter to ensure sufficient overlap of adjacent illuminated areas for a robust ptychographic reconstruction, in agreement with previous studies [55]. In the present case, this means that the illumination on the sample should have a diameter of at least 12 *µ*m, which is too large to meet the reciprocal-space sampling requirement given our experimental configuration. Conversely, near-field ptychography does not impose such sampling requirements related to the illumination size. We thus adjusted the focus-to-sample distance in such a way that the magnified image of the sample onto the detector had a pixel size approximately matching the aimed resolution of 100 nm. In this way, we could keep the overhead between acquisitions low while delivering the necessary fluence to the sample.

While the XNH measurement presented here is performed at a fourth-generation synchrotron source, the used photon flux was reduced by around two orders of magnitude to preserve the KB mirrors, resulting in a flux of 1.1 × 10^11^ photons/s on the sample mentioned in Section 4. Advances in instrumentation are needed to fully harvest the benefits of the latest-generation sources in terms of throughput. The acquisition in XNH can be further accelerated by using on-the-fly tomographic scanning [56], and by using photon-counting X-ray detectors, and X-ray mirrors compatible with the photon flux provided by the 4th generation sources. The use of single-distance holography techniques may also help to reduce the acquisition time [57,58]. The PXCT measurement could benefit significantly from the increase of coherent flux at fourth-generation sources. Additional gains of up to two orders of magnitude might further be achievable through emerging technologies such as broadband monochromators and more efficient X-ray lenses [59], as broad-bandwidth ptychography shows promise for high-throughput, high-resolution imaging [60–62]. This increased coherent flux could be explored with the same experimental configuration reported here, using far-field ptychography, where the spatial resolution would be determined by the maximum angle at which scattered intensity by the sample can be reliably measured. However, for the current experimental parameters this would require reducing the size of the illumination to fulfill the minimum sampling condition for ptychography, requiring even more scanning positions to ensure the overlapping of the illumination at neighboring scanning positions and thus introducing additional overhead in the total acquisition time. Alternatively, one could retain a near-field implementation using a detector with a smaller pixel size. The detector used here for near-field ptychography has the advantage of being a single-photon counting detector with nearly 100% efficiency. Therefore, one could consider keeping such a detector and instead increasing the sample-detector distance to allow a smaller reconstructed pixel size matching the aimed resolution, or increase the numerical aperture of the illumination [63], while allowing a large illumination on the sample.

PXCT provides the 3D electron density distribution of the specimen with absolute quantitative contrast [48]. This is possible because all the 2D projections include sample-free regions, which can be used to correct the otherwise undetermined phase offset. Similarly, the amplitude projections provide quantitative 3D distributions of the linear attenuation coefficient of the specimen, not shown here. XNH provides as well the 3D electron density distribution inside the sample, albeit with lower accuracy. The precision of the quantitativeness in XNH can be improved by introducing reconstruction of the complex-valued probe function within the phase retrieval approach [64].

A comparison between XNH and near-field PXCT has been reported on a solid oxide cell sample of about 40 *µ*m diameter [65]. In that case, the sample structure presented a strong contrast between the different phases, with sharp interfaces between the different materials. For biological tissues, the contrast is lower even when EM metal staining protocols are used. Another difference to the work presented here is that in Ref. [65] both measurements were performed at the same instrument at the ID16A beamline at the ESRF using the same photon energy of 33.6 keV. Despite all these differences, this work also reveals a similar resolution between the two measurements and a faster measurement using XNH.

## 7 Conclusion

We performed measurements with X-ray near-field ptychographic computed tomography and X-ray holographic nanotomography on the same metal-stained, resin-embedded brain tissue sample, in cryogenic conditions. Measurements were conducted at two different synchrotrons, with the sample maintained at liquid nitrogen temperature throughout. For this, we designed and implemented suitable specimen supports compatible with both instruments. We found that the two technologies provided 3D images of the tissue with similar contrast and resolution in the particular case which was studied. Both techniques benefit from the improved coherence and photon flux offered by fourth generation synchrotron sources. Advances in high-precision and high-speed actuators, radiation resistant X-ray optics, and detectors will enable to fully exploit the benefits of the latest generation sources and scale up these nanoimaging approaches by orders of magnitude. These X-ray nanotomography techniques offer promising capabilities for multiscale imaging of brain tissues and mapping of neural wiring at synaptic resolution [36,66].

## Appendix: dose estimation

Since absorption, and thus the absorbed energy, is measured, the main uncertainty when estimating the dose stems from the estimate of the sample mass [48]. We estimate the sample composition by assuming that the chemical solutions used for staining reach equilibrium within a sample composed of protein and resin. We thus obtain the following sample stoichiometry: C_60.90_H_74.54_Cl_1.46_O_15.15_N_1.08_Os_0.16_U_0.05_Pb_0.02_K_0.28_Fe_0.07_Ca_0.002_S_0.09_ [67]. This material composition corresponds to an average ratio of molecular weight to atomic number of *A*/*Z* = 1.90 mol / g, which is used in the conversion from electron density to mass density [48]. We note that the ratio *A*/*Z* does not change significantly for different sample compositions. For example, if we assume that the sample takes up five times more staining than assumed at equilibrium, we would obtain *A*/*Z* = 1.99 mol/g. We thus use an uncertainty for *A*/*Z* of 0.1 mol/g in our error estimation.

For the PXCT dataset, we obtain a dose *D*_a_ = (3.3 ± 0.5) × 10^7^ Gy. Due to the significantly higher photon energy, in the case of the XNH measurement we can apply the linear approximation, 1 − exp (−*µz*) ≈ *µz*, which yields the commonly used surface dose, $D_{\text{s}}=\frac{\mu }{\rho }N_{0}E$, where *µ* and *ρ* are the linear attenuation coefficient and the mass density of the sample material, *N*_0_ is the number of photons incident on the sample per unit area, and *E* is the X-ray photon energy [68]. We thus obtain *D*_s_ = (8.6 ± 0.5) × 10^7^ Gy. These results are reported in Table 1.

## Supplementary Material

Supplementary Document

## Figures and Tables

**Table 1:** Experimental parameters for the acquisition of datasets.

Parameters	PXCT	XNH
Photon energy (keV)	6.2	17
Beam flux density on sample (photons/s/ *μ*m^2^)	9.9 × 10^5^	1.7 × 10^7*a*^
Total fluence on sample (photons/*μ*m^2^)	4.8 × 10^9^	1.6 × 10^10^
sample thickness (*μ*m)	108 ± 5	108 ± 5
vertical field of view (*μ*m)	30	128
Total measured volume (*μ*m^3^)	2.7 × 10^5^	1.2 × 10^6^
Voxel size (nm^3^)	93.8^3^	40.03
FSC resolution (nm^3^)	108^3^	121^3^
Total acquisition time (h)	9.75	0.33
Total measurement time*^b^* (h)	15.75	3.1
Total processing time (h)	17	3
Measurement speed (voxels/s)	5.9 × 10^3^	1.7 × 10^6^
Measurement speed (resels*^c^*/s)	3.8 × 10^3^	6.1 × 10^4^
Attenuation length*^d^ (μm)*	69	370
Estimated dose (Gy)	(3.3 ± 0.5) × 10^7^	(8.6 ± 0.5) × 10^7^

a For the shortest focus-sample distance.

b Including overhead time, e.g. due to movement of stages between acquisitions.

c Resels denotes resolution elements, where the FSC resolution has been used.

d Defined as the material thickness at which X-rays are attenuated by 1/*e*. Attenuation length values were obtained from X-ray transmission measured through the center of the sample.

## Data Availability

The raw data corresponding to the PXCT dataset is available under [69], and the corresponding derived data, including ptychographic reconstructions of all projections and the final 3D tomographic data volume, is available under [70]. The raw and reconstructed data acquired at ESRF will be made available at [71].
