# Supplementary material for: Experimental comparison of X-ray ptychographic and holographic nanotomography of metal-stained neuronal tissue
Source: Opt Express. Author manuscript; Available in PMC 2025 Oct 10. (PMC12329787; doi:10.1364/OE.563186)
Supplement: Supplementary Document [file EMS209505-supplement-Supplementary_Document.pdf]

Supplemental Document

Optics EXPRESS

Experimental comparison of X-ray ptychographic and holographic nanotomography of metal-stained neuronal tissue: supplement

ALEXANDRA PACUREANU,[1] THOMAS THIES,[2] CARLES BOSCH,[3] MIRKO HOLLER,[2] MANUEL GUIZAR-SICAIROS,[2,4] ELISABETH MÜLLER,[2] JOAKIM REUTELER,[5] DMITRY KARPOV,[1] ANDREAS T. SCHAEFER,[3] PETER CLOETENS,[1] ANDREAS MENZEL,[2] AND ANA DIAZ[2,*]

[1] *ESRF, the European Synchrotron, 71 Av. des Martyrs, 38000 Grenoble, France*
[2] *Paul Scherrer Institute, Forschungsstrasse 111, 5232 Villigen-PSI, Switzerland*
[3] *Francis Crick Institute, 1 Midland Rd, London NW1 1AT, United Kingdom*
[4] *École Polytechnique Fédérale de Lausanne (EPFL), 1015 Lausanne, Switzerland*
[5] *ScopeM, ETH Zurich, Switzerland*
[*] *ana.diaz@psi.ch*

This supplement published with Optica Publishing Group on 16 June 2025 by The Authors under the terms of the Creative Commons Attribution 4.0 License in the format provided by the authors and unedited. Further distribution of this work must maintain attribution to the author(s) and the published article's title, journal citation, and DOI.

Supplement DOI: https://doi.org/10.6084/m9.figshare.29232251

Parent Article DOI: https://doi.org/10.1364/OE.563186

Supplementary Materials

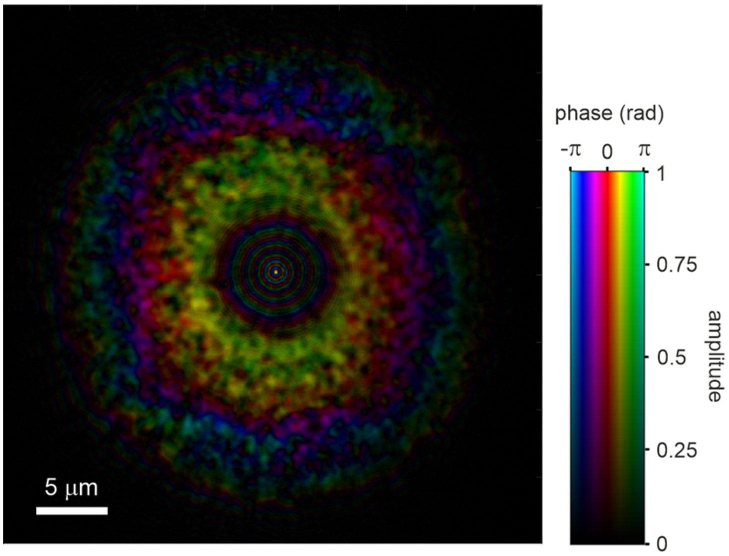

Figure S1: reconstructed probe from the same projection as that shown in Fig. 1 in the main text. The complex-valued probe is shown in a color scale where the hue and the value represent the phase and the amplitude, respectively. The illumination on the sample has a diameter of about 30 microns and it is structured due to the use of a Fresnel zone plate (FZP) with locally misplaced zones, as described in Ref. (Odstrcil *et al.*, 2019). This type of aberrated FZP is specifically designed to produce an optimized illumination for ptychography, resulting in faster convergence, specially in cases where the sample does not have a strong structure, or when it features phase shifts spanning many multiples of 2π, as in our case, as shown in Fig. 1(c).

(Odstrcil *et al.*, 2019) M. Odstrčil, M. Lebugle, M. Guizar-Sicairos, C. David, and M. Holler, "Towards optimized illumination for high-resolution ptychography," Opt. Express **27**, 14981–14997 (2019).

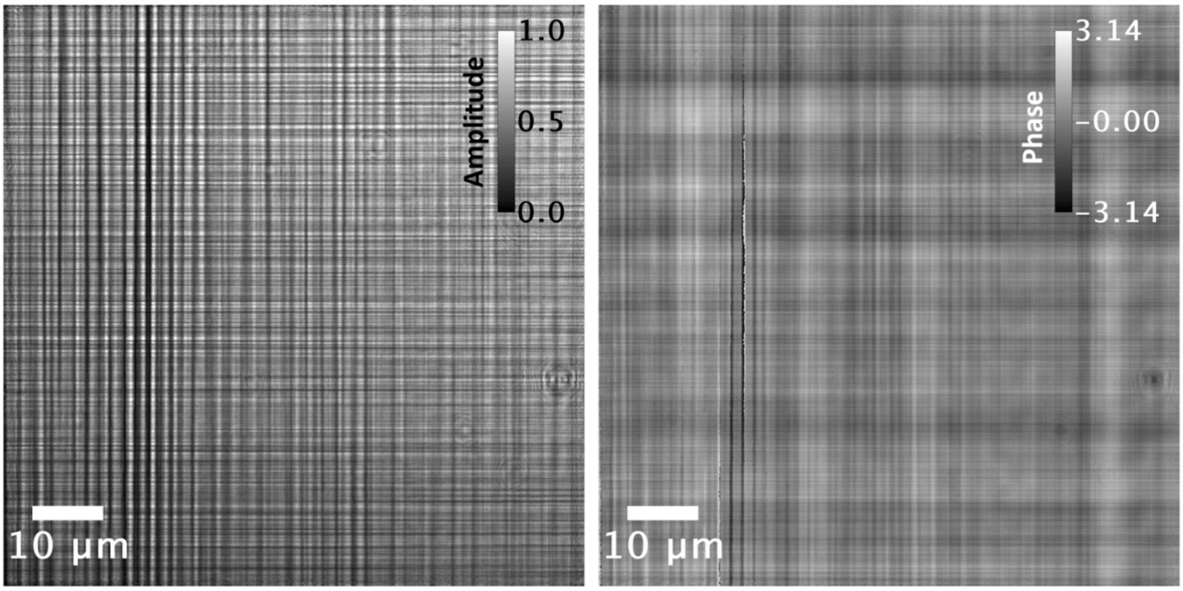

Figure S2: The amplitude and phase of the probe used for the XNH scan. The reconstruction is based on a near-field ptychographic acquisition (Stockmar et al., 2013), recorded just before the XNH scan. The reconstruction was performed using the PyNX library (Favre *et al.*, 2020).

(Favre *et al.*, 2020) V. Favre-Nicolin, G. Girard, S. Leake, J. Carnis, Y. Chushkin, J. Kieffer, P. Paleo, M.-I. Richard, “PyNX: high-performance computing toolkit for coherent X-ray imaging based on operators”, Appl. Crystallog. **53**, 1404-1413 (2020).
